# Structural brain network characteristics in patients with episodic and chronic migraine

**DOI:** 10.1186/s10194-021-01216-8

**Published:** 2021-03-03

**Authors:** Lars Michels, Nabin Koirala, Sergiu Groppa, Roger Luechinger, Andreas R. Gantenbein, Peter S. Sandor, Spyros Kollias, Franz Riederer, Muthuraman Muthuraman

**Affiliations:** 1 Department of Neuroradiology, University Hospital Zurich, Sternwartstr. 6, CH-8091 Zurich, Switzerland; 2 Haskins Laboratories, New Haven, Connecticut USA; 3 Section of Movement Disorders and Neurostimulation, Biomedical Statistics and Multimodal Signal Processing unit, Department of Neurology, Focus Program Translational Neuroscience (FTN), University Medical Center of the Johannes Gutenberg-University Mainz, Mainz, Germany; 4 Institute for Biomedical Engineering, University and ETH Zurich, Zurich, Switzerland; 5 Department of Neurology and Neurorehabilitation, RehaClinic, Bad Zurzach, CH-5330 Switzerland; 6 Department of Neurology, University Hospital Zurich, CH-8091 Zurich, Switzerland; 7 Department of Neurology, Clinic Hietzing and Karl Landsteiner Institute for Clinical Epilepsy Research and Cognitive Neurology, Wolkerssbergenstrasse 1, AT-1130 Vienna, Austria; 8 University of Zurich, Faculty of Medicine, Rämistrasse 100, CH-8091 Zurich, Switzerland

**Keywords:** Migraine, Episodic, Chronic, Graph theory, Connectivity

## Abstract

**Background:**

Migraine is a primary headache disorder that can be classified into an episodic (EM) and a chronic form (CM). Network analysis within the graph-theoretical framework based on connectivity patterns provides an approach to observe large-scale structural integrity. We test the hypothesis that migraineurs are characterized by a segregated network.

**Methods:**

19 healthy controls (HC), 17 EM patients and 12 CM patients were included. Cortical thickness and subcortical volumes were computed, and topology was analyzed using a graph theory analytical framework and network-based statistics. We further used support vector machines regression (SVR) to identify whether these network measures were able to predict clinical parameters.

**Results:**

Network based statistics revealed significantly lower interregional connectivity strength between anatomical compartments including the fronto-temporal, parietal and visual areas in EM and CM when compared to HC. Higher assortativity was seen in both patients’ group, with higher modularity for CM and higher transitivity for EM compared to HC. For subcortical networks, higher assortativity and transitivity were observed for both patients’ group with higher modularity for CM. SVR revealed that network measures could robustly predict clinical parameters for migraineurs.

**Conclusion:**

We found global network disruption for EM and CM indicated by highly segregated network in migraine patients compared to HC. Higher modularity but lower clustering coefficient in CM is suggestive of more segregation in this group compared to EM. The presence of a segregated network could be a sign of maladaptive reorganization of headache related brain circuits, leading to migraine attacks or secondary alterations to pain.

**Supplementary Information:**

The online version contains supplementary material available at 10.1186/s10194-021-01216-8.

## Introduction

Migraine is a multifactorial neurovascular disorder which affects about 12% of the general population [1] and rates among the most disabling diseases [2, 3]. In the episodic form of migraine (EM), headache occurs less than 15 days per month, whereas in the chronic form (CM), it occurs on 15 or more per month for at least three consecutive months [4]. Apart from functional brain alterations [5–8], several [9–15] but not all studies [16] reported multi-regional volumetric alterations in white mater and gray matter. Grey matter volume (GMV) alterations can get worse (i.e. seen as decreases in GMV) over time, e.g. in sensory-discriminative brain regions [10], although the existences of longitudinal changes in GMV are still under debate [17, 18]. Regarding the spatial locations of migraine-related GMV changes, previous studies reported alterations in the occipital [14], frontal [8, 11, 14, 19–21], temporal cortex [9], somatosensory [22], parietal cortex [19, 20] and cerebellar regions [15]. Similarly, other studies demonstrated altered cortical thickness (CT) in migraineurs compared to controls, seen in the visual cortex [23–25], somatosensory cortex [22], frontal cortex [26], and temporo-parietal cortex [27].

However, a different analysis framework is required - moving away from regional GMV or CT differences – to assess the inter-relation between the described brain regions on a network level. This approach would allow relating GMV connectedness and integrity to symptom severity (i.e., EM and CM) and would thus allow a systematic and integrative way to analyze structural abnormalities in patients with migraine. In particular, a network analysis using a graph theoretical framework has been extensively used for observing effect of various disorders in brain network integrity. This framework considers brain regions as nodes, and the interrelations between them as edges to form a network [28, 29]. The network formation begins with the collection of relational data among elements of a neurobiological system, which may vary from anatomical networks of associations between morphometry of cortical regions, inter-regional white matter projections, or multi-dimensional time series and their statistical dependencies or causal relations in behavior in social interactions. Once this data is corrected, normalized and assembled into the mathematical form of a graph or network, the common mathematical framework of graph theory is applied to obtain a set of measures to observe different alterations in the network [30]. Using the anatomical networks reconstructed using the gray matter volumes (GMV) and functional network using resting-state functional magnetic resonance imaging (rs-fMRI)-correlations, Liu and colleagues demonstrated that network characteristics were disrupted in females with EM [31]. From the networks from diffusion tensor imaging (DTI) and rs-fMRI it has been shown that CM exhibited altered rich club organization (higher connection density, abnormal small-world organization with increased global efficiency) compared to healthy controls (HC). It was further concluded that the higher ‘bridgeness’ in patients with non-rich club regions might increase the integration among pain-related brain circuits with more excitability but less inhibition for the modulation of migraine [32]. In summary, the graph theory findings indicate that migraineurs lose structural network integrity, most likely seen as maladaptive integration among pain-related brain circuits, resulting in a disturbed balance of neuronal excitation and inhibition.

Yet, a systematic comparison of structural morphometric measures complimented with the brain network analysis has not been performed between patients with low (EM) and high (CM) occurrence of monthly migraine attacks. We thus examined cortical and subcortical morphometric changes leading to brain network reorganization in both patients with EM and CM relative to HC. Based on extant findings, we hypothesize to see the strongest global structural network alterations, seen as dis-integrated networks in patients with CM compared to HC and EM.

## Methods

### Design and study duration

This is the primary analysis of the reported data using a cross-sectional design. Other imaging data (MR spectroscopy (MRS) and Arterial Spin Labeling (ASL) MR imaging) have been collected for all participants and results are presented elsewhere [33]. No statistical power calculation was conducted prior to the study. The sample size was based on the available data (during the study interval) and was similar to a recent ASL study in episodic migraine patients [34]. All data was collected between December 2013 and July 2015.

### Participants

19 right-handed HC, 17 right-handed patients with EM and 12 patients with CM were included for the study. The detailed demographic data are listed in Table 1. All patients fulfilled the modified ICHD-III-beta diagnostic criteria for EM or CM [35]. None of the HC demonstrated signs of EM or CM according to these criteria (family history of migraine was allowed). During the enrollment process, we excluded all patients, which suffered from comorbid tension-type headache. Six of 12 CM also fulfilled the criteria for medication overuse headache (MOH), which is line to the literature [36, 37]. MOH is defined as headache that develops or significantly worsens during overuse of acute pain medication [35]. For all participants, exclusion criteria were severe psychiatric disorders, cardiac problems (e.g. severe hypertension), other headache disorders or other neurologic disorders such as epilepsy, stroke, traumatic brain injury, neck injury or cerebrovascular disease. All participants completed prospective headache diaries, the Migraine Disability Assessment (MIDAS) [38] and Hamilton Anxiety (HADS-A) and Depression (HADS-D) Score [39] questionnaires. Acute and prophylactic medication was recorded prior to the study interval for each patient (see Table 2). We assessed the attacks/month based on the MIDAS questionnaire. Here, the label “headache attacks/month rate” (Table 1) reflects the average number of migraine headache days in the last three months prior to the MRI (i.e., an attack frequency of 4.0 in EM means that the average number of headache days was four per month across in this group). We recorded aura occurrence in all patients electronically in a table. Patients were free from migraine attacks at least 48 h before and after the scan. The study was approved by the ethics committee of canton Zurich (KEK number E-37/2007), Switzerland. All subjects provided written informed consent prior to study enrolment. Both groups received 50 Swiss Francs reimbursement for their study participation. Patients were recruited by advertisement (Intranet of the Hospital and mailing lists) and word-of-mouth.

**Table 1:** Demographic details of the subjects in the study

Group	N	Age (years)*	Sex*	MIDAS	HADS - A	HADS - D	Headache attacks / month*
EM	17	32.7 ± 9.9	F = 13, M = 4	19.65 ± 20.61	5.3 ± 3.9	3.4 ± 2.6	4.0 ± 3.8
CM	12	38.19 ± 16.15	F = 8, M = 4	55.50 ± 12.76	5 ± 3.46	4.67 ± 2.96	18.50 ± 4.25
HC	19	31.7 ± 9.2	F = 10, M = 9	N/A	3.4 ± 2.3	1.3 ± 1.2	N/A
EM/CM with aura	12EM/5CM	35.68 ± 13.15	F = 12, M = 5	30.47 ± 28.32	5.11 ± 3.55	3.88 ± 2.69	8.64 ± 8.71

* We found no significant difference (*p* > 0.05) in age and sex between the groups: EM - HC, CM - HC and EM – CM (unpaired t-tests and Chi-Square test, respectively). MIDAS values in days. *EM* episodic migraine, *CM* chronic migraine, *HC* healthy controls, *F* female, *M* male.

* The label “headache attacks/month rate” represents the average number of migraine headache days in the last three months prior to the MRI (i.e., an attack frequency of 4.0 in EM means that the average number of headache days was four per month across in this group)

**Table 2:** List of the preventive (prophylaxis and acute medication) therapy for each patient

EM	Acute	Prophylactic	CM	Acute	Prophylactic	MOH
Subj. 1	SA		Subj. 1	SA	B2, Mg	no
Subj. 2	SA		Subj. 2	Triptans		yes
Subj. 3	Triptan		Subj. 3	Triptans, SA		yes
Subj. 4	Triptan, SA		Subj. 4	Triptans		no
Subj. 5	SA		Subj. 5	Triptans		no
Subj. 6	Triptans		Subj. 6	Triptans		yes
Subj. 7	Triptans		Subj. 7	Triptans	Betablocker	yes
Subj. 8	SA, opiates		Subj. 8	Triptans, SA		yes
Subj. 9	SA		Subj. 9	SA		no
Subj. 10	SA^a^		Subj. 10	Triptans	Riboflavin	yes
Subj. 11	SA		Subj. 11	SA		no
Subj. 12	SA		Subj. 12	SA		no
Subj. 13	SA^a^					
Subj. 14	Triptans, SA					
Subj. 15	SA^a^					
Subj. 16	SA					
Subj. 17	Triptans, SA	B2, Mg, Q10				

*Abbreviations*: *EM* Episodic migraine, *CM* Chronic migraine, *SA* simple analgesics, *SA* ^a^ - simple analgesics (not for every attack), *B2* Riboflavin, *Mg* Magnesium, *Q10* coenzyme Q10, *MOH* medication overuse headache. MOH is defined as headache that develops or significantly worsens during overuse of acute pain medication

### Data acquisition

Whole-brain magnetic resonance imaging (MRI) was performed on a 3 T scanner (Philips Ingenia, Netherlands) with a 32-channel receive-only head coil at the Neuroimaging Center of the University Hospital Zurich. 3D T1-weighted magnetization prepared rapid gradient echo (MPRAGE) sequence was acquired for each subject with TE/TI/TR = 2.52/900/1900 ms, flip angle = x°, field of view (FOV) = 256 × 256 mm^2^, matrix size = 256 × 256, slab thickness = 192 mm, voxel size = 1 × 1 × 1 mm^3^. Subjects’ scans were examined for any major anatomical abnormalities by an experienced neuroradiologist.

### Data analysis

#### Cortical and subcortical morphometric analysis

Data from all subjects were analyzed using FreeSurfer version 5.3.0 (http://surfer.nmr.mgh.harvard.edu). This automated anatomic parcellation procedure enables one to extract reliable estimates of various cortical and subcortical measures including thickness, volume, area, curvature etc. [40]. The procedure includes several steps: intensity normalization, skull stripping, Talairach transformation, and atlas-based assignment of neuro-anatomical labels, which are described in detail in previous studies [41, 42]. To describe here briefly, all subjects were run using the “recon-all” processing stream with default parameters to create a cortical surface model. This process includes above-mentioned procedures along with motion correction, averaging of multiple T1 volumes, removal of non-brain tissue and grey matter white matter boundary tessellation to create the surface model. This obtained model is then further used with its intensity and continuity information from the entire 3D volume in segmentation and deformation procedures to generate cortical thickness, calculated as the closest distance from the gray/white boundary to the gray/CSF boundary at each vertex on the tessellated surface [41]. This process of obtained morphometric measures have been validated using histological [43] and manual measurements [44] and have been demonstrated to have very high reliability across different scanner and field strengths [45]. These cortical and subcortical morphometric measures are very effective in depicting the regional alterations. However, the effect of these regional changes to other associated regions could have a significant impact in overall information transfer leading to various functional modifications. Hence, to observe these network level differences between the groups the computed cortical thickness (CT) and subcortical volumes (SCV) from all the subjects were further processed using brain network analysis.

#### Brain network analysis

Graph theoretical measures of network modularity, distance, and local information transfer was computed using the CT and subcortical volumes obtained from FreeSurfer using Brain Connectivity Toolbox [46] (https://sites.google.com/site/bctnet/). The group level correlations between the cortical regions and subcortical volumes and the differences between them were then computed in different network densities for observing the steady topological changes [47, 48]. The details of the analysis have been explained elsewhere [49, 50].

**Fig. 1:**
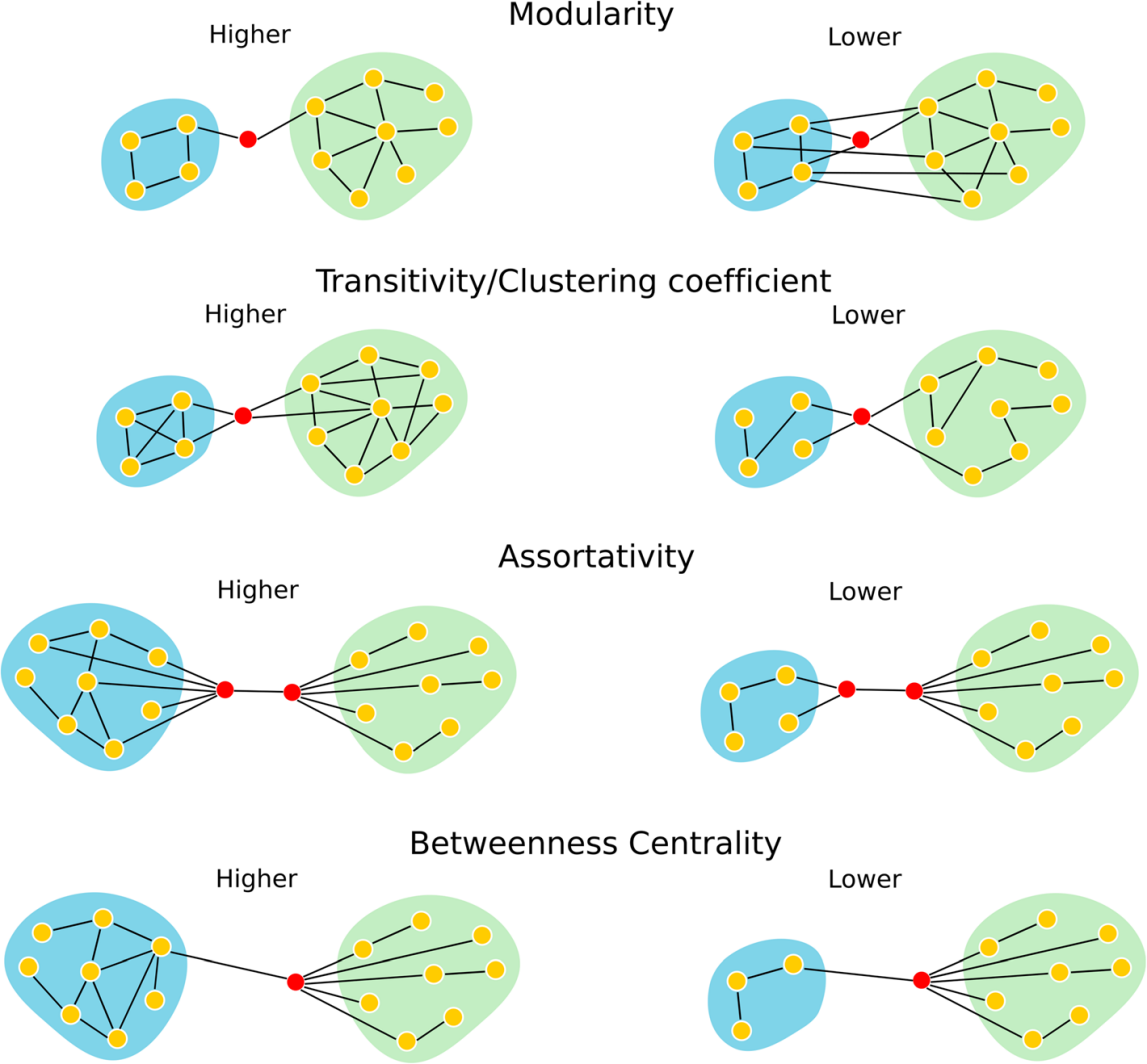
Simplistic illustration of the network measures used in the study. Higher assortativity illustrating the tendency of a high degree node to be connected to another higher degree node; higher transitivity shows the greater number of connections within the module (forming triangles, higher modularity illustrates the reduced inter-module connection between the network, and higher betweenness centrality displays node connecting two networks with large number of within module connections highlighting the importance of this node in the network

Among different measures computed in the study, below is the brief overview of those relevant for the study, with simplistic illustration in Fig. 1.

- Modularity is a measure of the degree, to which the network is subdivided into densely interconnected nodes (modules) with sparse connections to other network or modules. Louvain algorithm was used for computing the modularity which is a hierarchical clustering algorithm, that recursively merges communities into a single node and executes the modularity [51].
- Transitivity is the ratio between the number of triangles and the number of triplets in the graph.
- Assortativity is correlation coefficient between the degrees of all nodes on two opposite ends of a link, higher (positive) assortativity indicating the nodes tend to link to other nodes with the same or similar degree.
- Clustering coefficient is the fraction of triangles around a node representing the node’s neighbors that are also neighbors of each other.
- Betweenness centrality of a node is the fraction of all shortest paths in the network that contain a given node. A node with higher edge-betweenness centrality participates in a large number of shortest paths.

In addition, network-based statistic (NBS) was used to assess differences in the inter-regional connectivity between the groups. NBS analysis performs the mass-univariate testing at every connection comprising the graph controlling for multiple comparisons through evaluating the null hypothesis at the level of interconnected subnetworks rather than individual connections [52]. Here, the connectivity matrices obtained from the association of CT and SCV between the regions across a range of network densities were subjected to NBS analysis. The analysis primary goal was to identify the sub-network with the regions shown to have significant difference in various network properties using graph theoretical measures. Further details regarding the procedure are mentioned elsewhere [53, 54].

#### Statistical analysis

For the graph theoretical framework analysis, we used the CT values of a network comprising 34 cortical regions in each hemisphere based on Desikan atlas [55]. Subsequently, we used the sub-cortical volumes from nine regions in each hemisphere and brain stem for the sub-cortical network. For assessing the statistical significance of graph metrics between patients and HC, a nonparametric permutation tests with 5000 iterations were applied [56, 57]. Given that, CT is sensitive to age and sex, they were further used as covariates for the analysis. In each repetition, the regional data for each subject were randomly reassigned to one of the two groups and an association matrix was obtained. The network measures were then calculated for all the networks at each density. Here, density represents cost of the network computed by fraction of present connections to all possible connections. Hence, the network measures derived at each density would specify the alterations in network behavior at different levels of fragmentation (from full, partial to discontinuous connectivity). This method of thresholding ensures that all the regions (nodes) of the network are connected while discarding spurious connections (edges) [47, 58]. The actual between-group difference in network measures was then placed in the corresponding permutation distribution and a two-tailed *p*-value (at 5% significance level, false discovery rate (FDR) corrected) was calculated based on its percentile position [59].

To assess the statistical significance for CT correlations with different clinical parameters, QDEC – a FreeSurfer statistical toolbox was used. Here, surface maps depicting regions with significant differences in the correlation with CT at each vertex were determined with general linear models (GLMs) using *p* < 0.001 as the threshold for a significant cluster. In addition, we further performed the GLM analysis to observe the association between the CT change and different clinical scores including HADS-A, HADS-D, hours of sleep and attacks per month.

To validate the significance of these network measures, we further applied support vector machine analysis to predict the clinical scores used in the diagnostic criteria for migraineurs. Here, we performed a support vector regressor (SVR) analysis – representing a machine-learning-based multiple regression method - that could associate the observed and trained values and present the regression coefficient for the accuracy of the prediction [60]. The regression coefficient of 0.5 obtained after 10-fold cross validation is considered borderline significant result.

## Results

### Structural network analysis

**Fig. 2:**
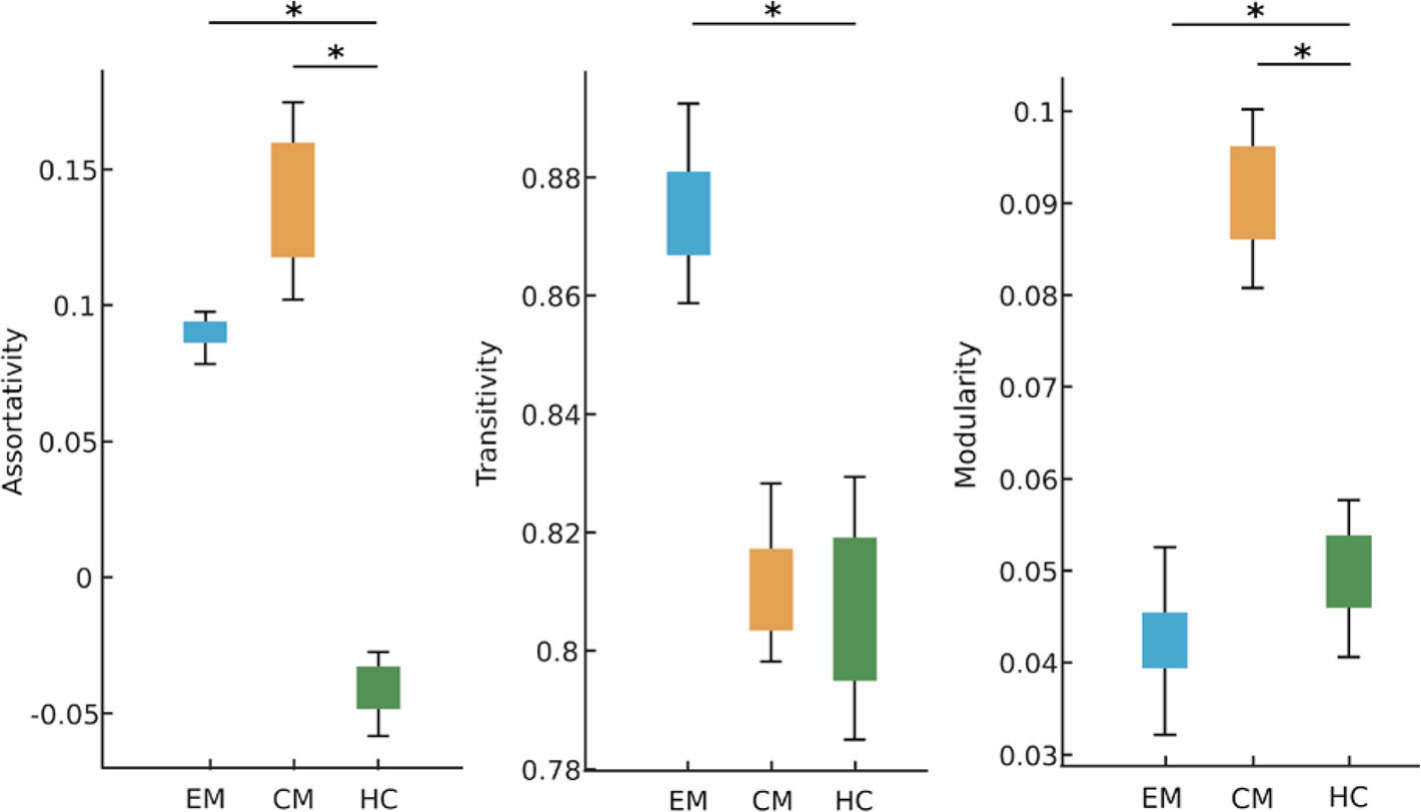
Cortical network results: Plots showing the network measures (assortativity, transitivity and modularity) significantly different between the groups: episodic migraine patients (EM), chronic migraine patients (CM) and healthy controls (HC) obtained using CT. * indicates significant group differences at *p* < 0.05 (FDR corrected)

We found no significant difference (*p* > 0.05) in age and sex between the groups: EM - HC, CM - HC and EM - CM. Comparing networks obtained using graph theoretical framework between EM and HC, we found significantly (*p* < 0.05, FDR corrected) higher transitivity and assortativity in EM (Fig. 2). We further obtained the centrality measures, namely mean node and edge-betweenness to be higher in EM. For the contrast ‘CM – HC’, we found significantly higher modularity and assortativity in CM. We further obtained higher centrality measures (mean node and edge-betweenness) in CM. For the comparison of the networks between two patient groups, we found significantly higher modularity but lower clustering coefficient and transitivity in CM compared to EM.

**Fig. 3:**
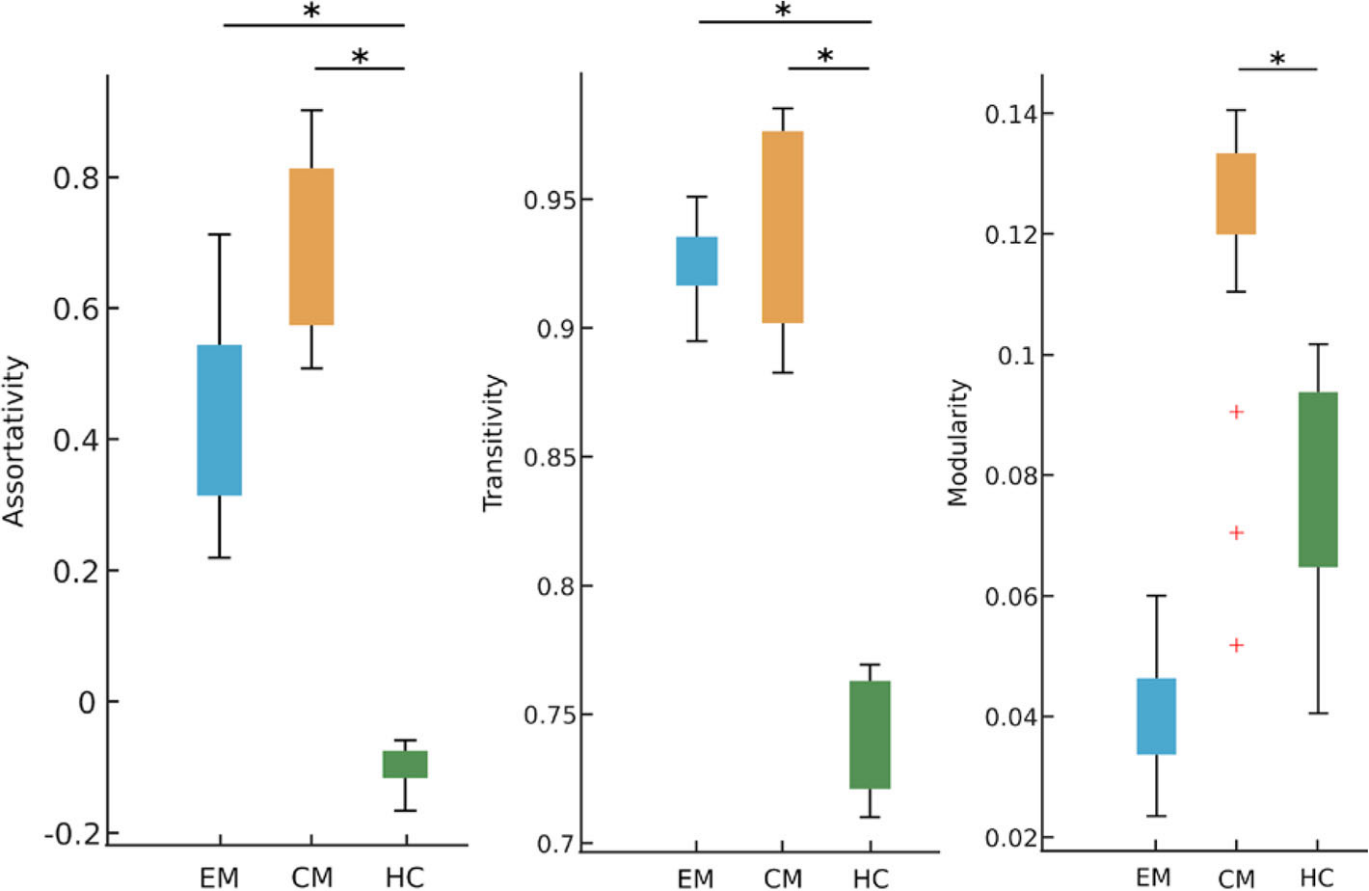
Sub-cortical network results: Plots showing the network measures (assortativity, transitivity and modularity) significantly different between the groups: episodic migraine patients (EM), chronic migraine patients (CM) and healthy controls (HC) obtained using sub-cortical volumes. * indicates significant group differences at *p* < 0.05 (FDR corrected) and ^+^ indicates outliers (i.e. 2 standard deviations from the groups’ mean)

When comparing similar networks obtained using values of subcortical volumes between EM and HC, we found higher transitivity and assortativity significantly in EM. The comparison CM and HC revealed significantly higher modularity, transitivity and assortativity in CM. All sub-cortical network results are shown in Fig. 3.

**Fig. 4:**
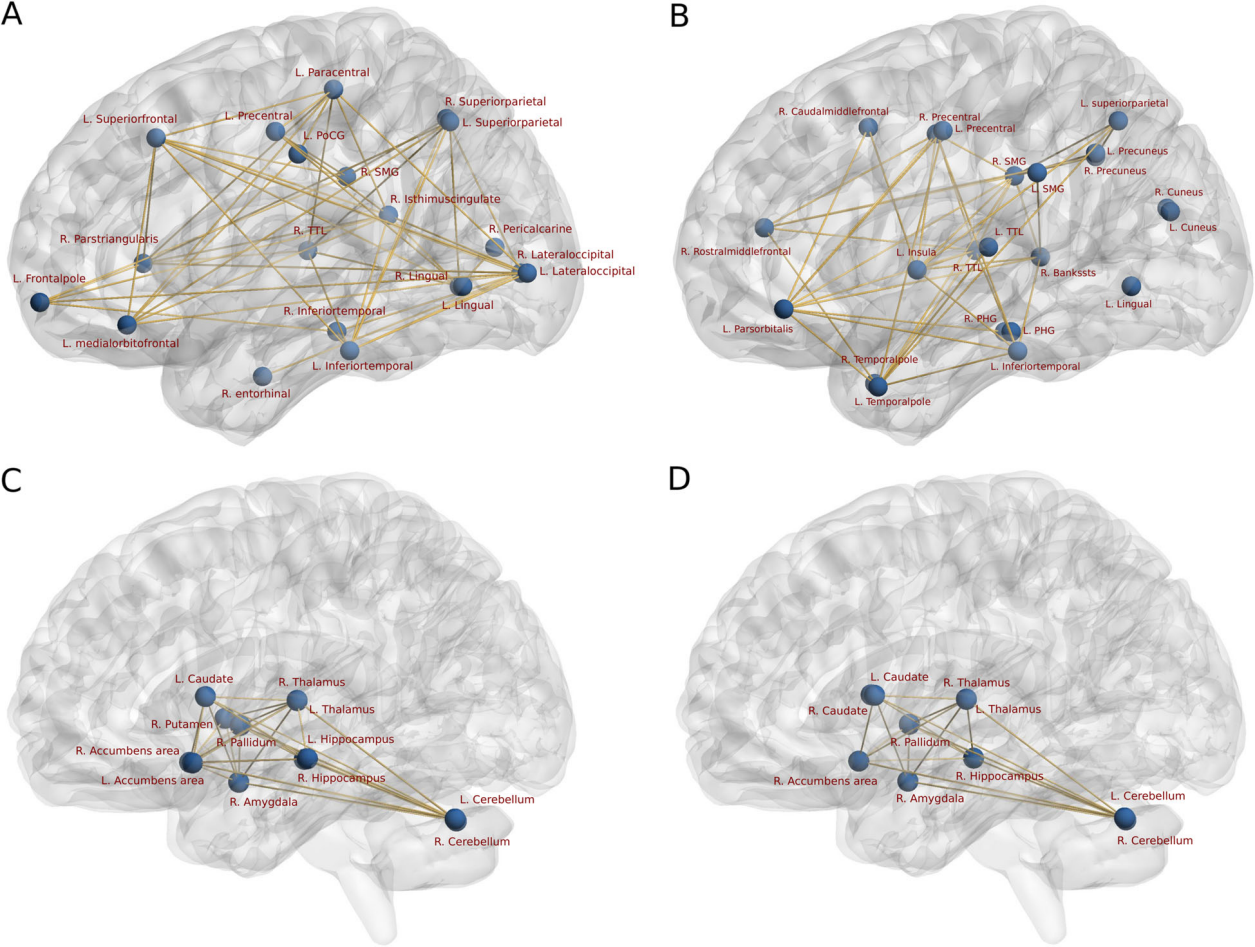
(A & B) Cortical network (obtained using cortical thickness) showing significant (p < 0.05, corrected) lower structural connectivity in Episodic (A) and Chronic (B) migraine patients in comparison to HC. Abbreviations: PoCG – Posterior cingulate gyrus, TTL – Transverse temporal lobe, PHG – Parahippocampal Gyrus, SMG – Supramarginal Gyrus. L - Left and R – Right hemisphere. (C & D) Subcortical network (obtained using subcortical volumes) showing significant (p < 0.05, corrected) lower structural connectivity in Episodic (C) and Chronic (D) migraine patients in comparison to HC

**Fig. 5:**
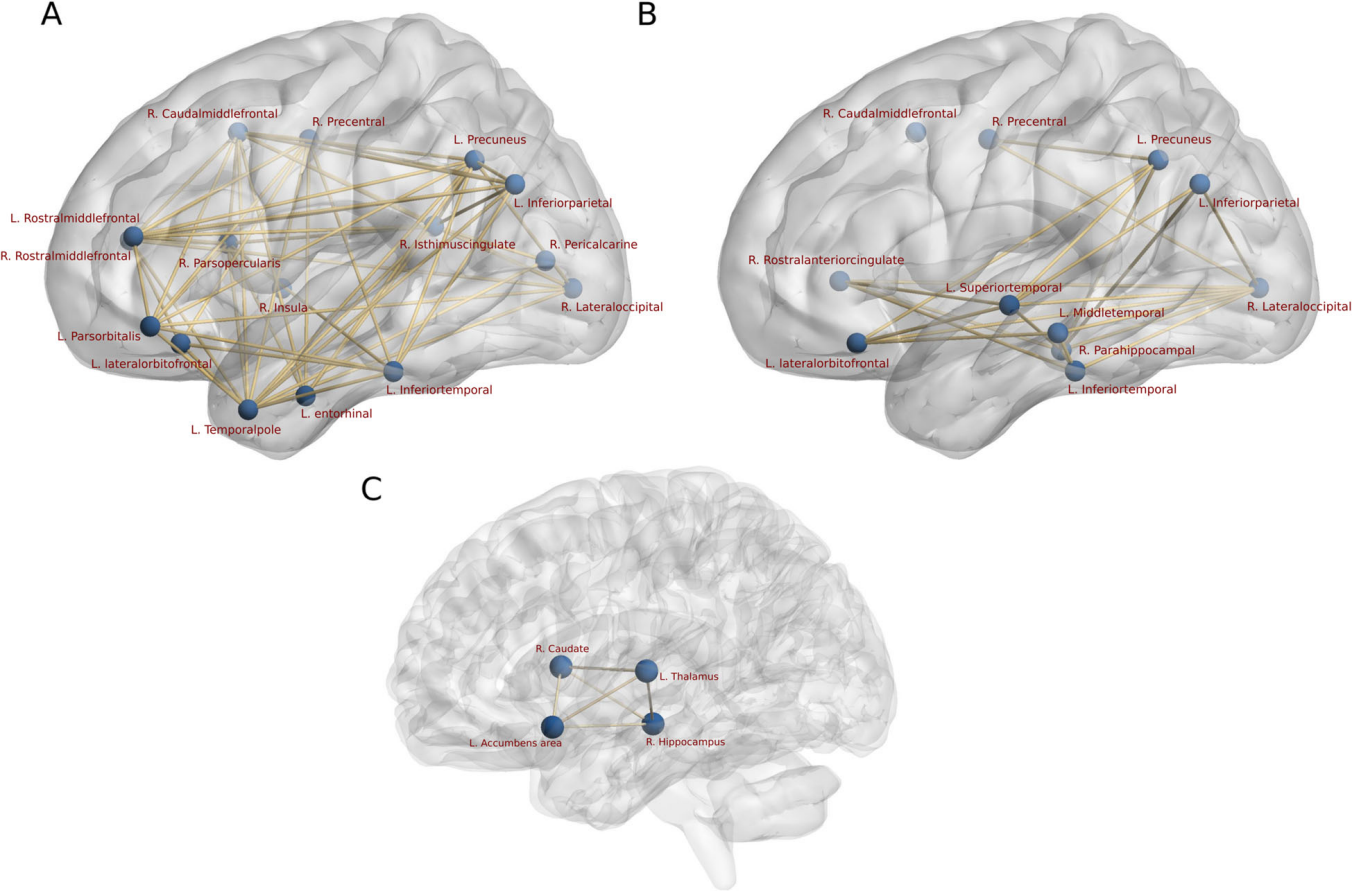
(A & B) Cortical network (obtained using cortical thickness) showing significant (*p* < 0.05, corrected) lower structural connectivity in Chronic than Episodic [CM < EM] (A) and Episodic than Chronic [EM < CM] (B) migraine patients. (C) Subcortical network (obtained using subcortical volumes) showing significant (*p* < 0.05, corrected) lower structural connectivity in Episodic than in Chronic [EM < CM] migraine patient. No regions were significant for [CM < EM]

Among the cortical regions showing significant regional network difference in terms of clustering, degree and nodal edge-betweenness obtained using CT, NBS further revealed distinct networks with lower interregional connectivity for EM and CM when compared to HC. For EM, all 20 nodes (regions) showing the graph theory differences formed a network of significantly (*p* < 0.05, corrected) reduced connectivity in comparison to HC (Fig. 4a). However, for CM, out of 22 nodes (regions) only 19 formed a network of significantly (*p* < 0.05, corrected) reduced connectivity when compared to HC (Fig. 4b). For the comparison between two migraine groups EM and CM, NBS exhibited two distinct subnetworks for the contrast EM < CM and CM < EM (Fig. 5a, b). As expected, the contrast CM < EM showed lower structural connectivity between greater number of nodes than in contrast EM < CM (16 ROIs compared to 11 ROIs), indicating higher structural connectivity loss for chronic migraineurs than episodic.

Similarly, among the subcortical regions showing the graph theoretical difference in measures - clustering, degree and nodal edge-betweenness obtained using SCV, NBS revealed distinct networks for EM and CM with lower interregional connectivity when compared to HC. For EM, we revealed that out of 18 subcortical regions analyzed, 12 showed graph theoretical differences and formed a network with significantly reduced connectivity compared to HC (Fig. 4c). However, for CM, only 10 subcortical regions showed graph theoretical differences and exhibit a subnetwork with significantly reduced connectivity compared to HC (Fig. 4d). For the comparison of EM and CM, interestingly only the contrast EM < CM yielded marginally significant subnetwork with only four ROIs (Fig. 5c) and the contrast CM < EM was not significant.

**Fig. 6:**
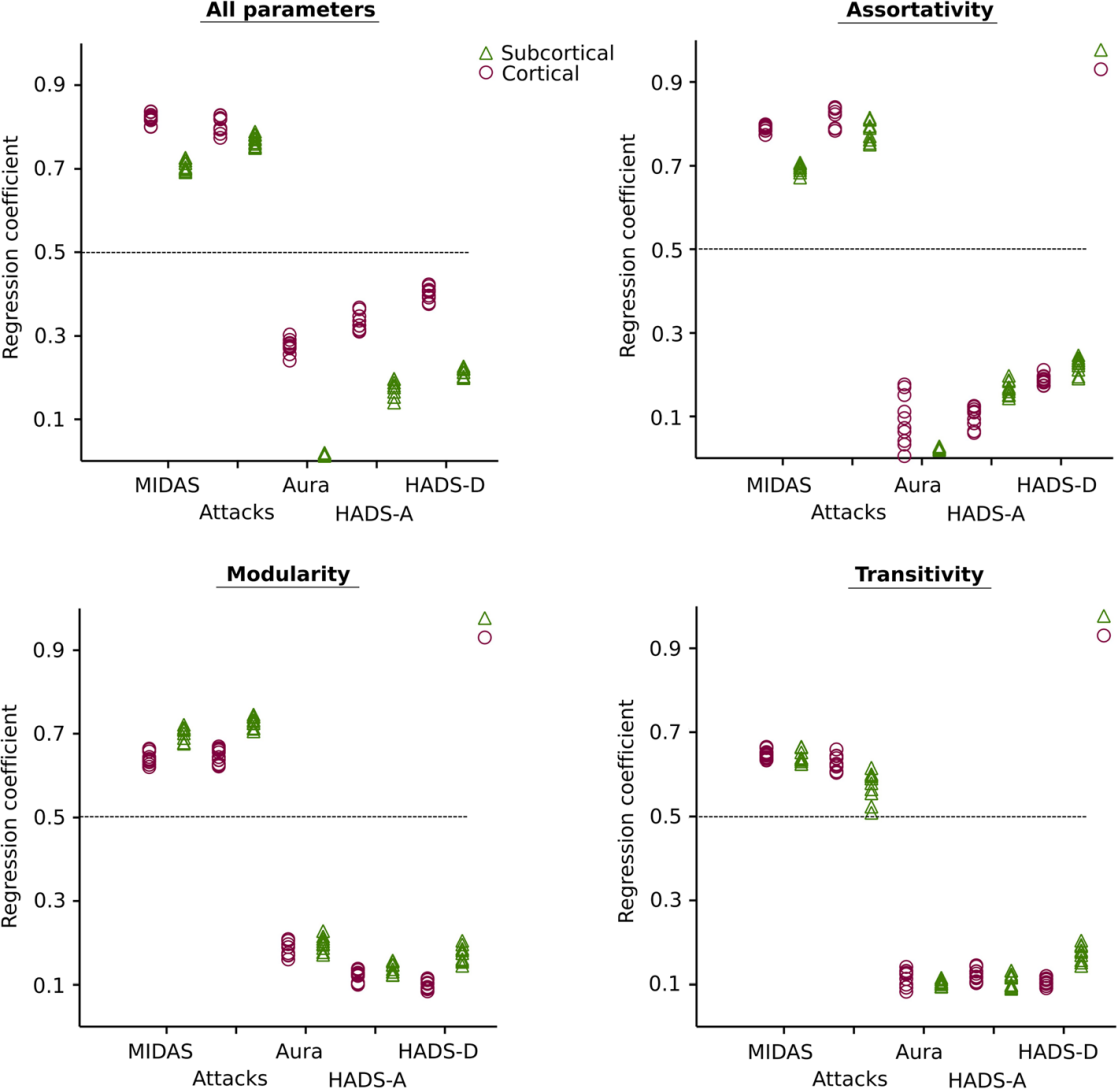
Plots showing the results of the SVR analysis, for predicting MIDAS, attacks per month, aura, HADS-A and HADS-D using network measures assortativity, transitivity and modularity obtained using cortical thickness and subcortical volumes. As the cross-validation was performed 10 times, each dot indicates the regression coefficient obtained for each validation. Dots above the dashed line indicate robust (r > 0.5) correlations

Finally, the network measures (assortativity, transitivity and modularity) obtained using both cortical thickness and subcortical volumes showing a significant difference between the groups, additionally yielded a significant interrelation for MIDAS and attacks per month for the migraineurs (Fig. 6). Considering all network measures together, the SVR yield MIDAS (across EM and CM) with a regression coefficient of 0.792 for CT, 0.712 for SCV and attacks with 0.798 for CT, 0.715 for SCV. Using only assortativity as network measure, the association for MIDAS and attacks was with a regression coefficient of 0.779 for CT, 0.690 for SCV and 0.812 for CT, 0.798 for SCV respectively. Similarly, for transitivity – MIDAS, it was 0.655 for CT, 0.647 for SCV and for transitivity – attacks, it was 0.649 for CT and 0.589 for SCV. Modularity alone could reveal the interrelation to the MIDAS and attacks with regression coefficient of 0.656 for CT, 0.698 for SCV and 0.649 for CT, 0.735 for SCV respectively.

### CT analysis

All reported CT group-differences and correlations were observed with age and sex as nuisance variables (*p* < 0.001, uncorrected as none of them survived multiple comparison (FDR) correction). Comparing EM to HC (**supp**. figure 1), average CT was significantly increased in EM in left lateral occipital cortex, supramarginal gyrus as well as in the right insula, lingual gyrus and precuneus. Additionally, the correlation of the average CT with HADS-A was significantly different in right precentral and inferior parietal cortex between EM and HC. Similarly, the correlation of the average CT with HADS-D was significantly different in left lingual gyrus and right supramarginal gyrus as well as the correlation of the average CT with hours of sleep in left superior parietal and right supramarginal gyrus and caudal middle frontal cortex between EM and HC.

The average CT was significantly increased in CM compared to HC in the left insula and posterior cingulate cortex (PCC) and significantly decreased in the bilateral inferior parietal and right lateral occipital cortex (**supp.** figure 2). Additionally, the correlation of the average CT with HADS-A was significantly different in right caudal anterior cingulate and precentral cortex between CM and HC. Similarly, the correlation of the average CT with HADS-D was significantly different in left lingual and PCC as well as the right supramarginal gyrus and precuneus between CM and HC. The correlation of the average CT with the hours of sleep differ significantly in left pars opercularis, superior parietal, insula and right lateral occipital and supramarginal gyrus.

The contrast ‘EM – CM’ (**supp.** figure 3) revealed significantly decreased average CT in EM in the left insula and significantly increased in right insula-, supramarginal- and postcentral gyrus. Additionally, the correlation of the average CT with HADS-A was significantly different in the bilateral inferior parietal and left superior parietal lobule. Similarly, the correlation of the average CT with HADS-D was significantly dissimilar in left insula between EM and CM. The correlation of the average CT with hours of sleep varied significantly in left superior parietal, insula and right supramarginal, postcentral and insula between EM and CM. Moreover, the correlation of the average CT with headache attacks per month was significantly different in left insula between these groups.

## Discussion

This study reported group differences in structural networks based on CT between HC, EM, and CM. Significant group differences were found in various graph theory measures and these measures were able to predict the clinical scores MIDAS and migraine attacks per month. Based on the results, we conclude that the impact of migraine severity leads to strong structural impairments and dysfunctional neural network configurations.

### Structural network alterations in migraineurs

Using both GMV and rs-fMRI data for reconstructing structural and functional connectivity, Liu et al. demonstrated that EM showed abnormal global topology in both structural and functional networks, characterized by higher mean clustering coefficients [31] . Even though these findings are noteworthy in aiding to comprehend the pathology, studies investigating structural topological changes, which might have led to these functional alterations and provide a probable explanation of symptoms in different types of migraineurs, are still distant. In our study, EM displayed higher transitivity and assortativity along with the centrality measures than HC, indicative of a shift in the hubs for information transfer in this group. Similarly, higher modularity, assortativity and centrality measures (mean node and edge-betweenness) in CM than in HC furthers indicates the network being more assortative and segregated in CM. This finding is additionally supported by the fact that CM were found to have significantly higher modularity but lower clustering coefficient and transitivity compared to EM. As the NBS analysis yielded lower interregional connectivity in migraineurs, we conclude that migraineurs display disturbed connections not only in a localized brain area but also between regions of different anatomical compartments including the frontal, temporal, parietal and visual areas.

Importantly, as clinically relevant, we demonstrated the importance of these network measures by being able to interrelate the MIDAS scores and attacks per month for the migraineurs. The interdependence between attack frequency and network measures was highest when using assortativity followed by all network measures (assortativity, transitivity and modularity) together. For MIDAS, using all three measures or the assortativity lead to a similar association. Hence, from these and previous findings we could speculate that there might be as well a transition from episodic to chronic with the intensification of network segregation and assortativity in migraineurs.

Out of 18 analyzed subcortical regions, 12 and 10 for EM and CM respectively, revealed graph theoretical differences and formed a network with significantly reduced connectivity compared to HC. Strikingly, the right caudate, left thalamus, left accumbens area and right hippocampus showed stronger alterations in CM compared to EM. This finding indicates that chronic migraine impairs structural network integrity not only on the cortical but also on the subcortical level.

### Brain morphometric alterations in migraineurs

Some studies reported CT increases in the somatosensory cortex [22] or higher visual brain regions, including V3A and MT+ [24], whereas others reported decreased CT in migraine patents with medication overuse headache [61] or in EM [25, 62, 63]. Our results indicate especially the CM showed a thicker CT compared to HC but that both EM and CM showed rather increases in CT, especially in visual brain regions (left occipital cortex, lingual gyrus). This could be related to the high presence of aura in our sample. Yet, Granziera et al., (2006) reported a CT increase in patients with and without aura in higher visual (V3A and MT+) brain regions. The CT increase could index altered excitability of the cortex in EM and even more so in CM. It has been shown that transcranial direct stimulation in EM can lead to reduced number of migraine days, indicating that neuronal excitation can be re-normalized resulting in lower migraine occurrence [64, 65]. Our novel finding of differentiating EM and CM based on association of CT and headache attacks observed in left insula underlines (a) the validity of CT as a structural marker to differentiate migraine subgroups and (b) the role of the salience network in migraine.

### Functional alterations in CM and MOH

Alterations in rs-fMRI have been demonstrated in several studies [66–72]. For example, Lee et al. (2019) applied rs-fMRI and observed increased functional connectivity of the pain matrix in CM [73]. Comparable to our findings, inter-regional coupling was altered in CM in a particular (pain) network comprising anterior insula, thalamus, prefrontal cortex, precuneus and anterior cingulate cortex. Using a seed-based approach, Schwedt et al. (2013) reported rs-fMRI connectivity differences between CM and controls in the anterior insula, amygdala, pulvinar, thalamus, middle temporal cortex, and periaqueductal gray (PAG) [70]. Our study adds that not functional connectivity shows a correlation to migraine frequency [70] but also graph theoretical measures. Recently, our group applied a network and a seed based (PAG) approach and found abnormal rs-fMRI connectivity in patients with MOH compared to patients with myofascial pain and healthy controls [72]. The alterations in MOH were seen as hyperconnectivity of the salience network (bilateral anterior insular cortex, dorsal anterior cingulate cortex, supplementary motor area), which correlated to white matter alterations in parts of this network (i.e. insular cortex), but also in the para-hippocampus, cerebellum and visual regions. As we observed alterations in structural connectivity in EM to HM in some of these regions (insula and anterior cingulate cortex), we cannot exclude that some of the observed effects related to CM might partially be mediated by the presence of MOH in some of the CM.

Some of the published rs-fMRI studies compared patients without aura to controls, and this sub-group comparison is not possible in our study, as the number of EM or CM without (and with) aura is too low. Yet, our SCV analysis extends rs-fMRI studies in CM as this group showed lower structural connectivity in EM and CM patients in comparison to HC in a network comprising amygdala, caudate nucleus, pallidum, thalamus, hippocampus and bilateral cerebellum. This observation is line to our previous study, which demonstrated structural (GMV) alterations in the basal ganglia (e.g., caudate nucleus and pallidum), hippocampus as well as in the thalamus, cerebellum, brainstem (PAG), visual and frontal cortex in patients with MOH [74]. Another group reported increases in GMV in the right amygdala and right putamen in CM compared to controls, and headache frequency correlated positively with GMV in the putamen as well as in frontal and temporal regions [75].

In our study, CM (compared to EM), showed structural alterations with a right-hemispheric dominance. In addition, the study by Chen et al. (2016) reported right-dominant alterations in rs-fMRI connectivity, i.e. between the marginal division of neostriatum and the right middle temporal or right middle frontal gyrus [67]. However, this was the case for EM, CM, and CM with MOH (compared to HC). Yet, a right dominance of GMV increased was also reported by a previous study in CM (some with MOH) [75].

## Limitations

It would have been interesting to examine the link between network alterations and patients’ disease duration, as done in a previous publication using resting-state fMRI [68]. However, this parameter could not be reliably assessed as many of the patients could just remember that they had migraine for “some years” or “since their adolescence or early adulthood”. Our group size was moderate, especially for the group of CM patients. Yet, we found a systematic increase in impairment on structural integrity with the presence of migraine attacks, indicating that CM caused the strongest structural abnormalities compared to HC and EM. The findings from CT and subcortical volume analysis were uncorrected which might be because of the moderate sample size. Nonetheless, the results present a significant basis for the network measures observed and gives a more enhanced tool for understanding the migraine pathophysiology.

## Conclusions

In conclusion, the level of impairment (migraine days per month) was associated with altered GVM but additionally with disturbed structural network integrity. The observation of an under-segregated network, especially in patients with CM, could be a sign of a maladaptive, elevated integration among pain-related brain circuits, leading to more excitability but less inhibition for the modulation of migraine.

## Supplementary Information

**Additional file 1 **: **Table S1.** Summary of significant regions as shown in supplementary figure 1. **Table S2.** Summary of significant regions as shown in supplementary figure 2. **Table S3.** Summary of significant regions as shown in supplementary figure 3.

**Additional file 2 **: **Figure S1.** Illustration of between-group differences for CT. (A) Regions depicting significant difference in the CT between EM and HC. Regions depicting significant difference between EM and HC in the correlation of average CT with HADS-A (B), HADS-D (C), and hours of sleep (D). A summary of the significant regions is reported in supplementary Table 1. All results are shown at *p* < 0.001 (uncorrected).

**Additional file 3 **: **Figure S2.** Chronic migraine patients (CM) and healthy controls (HC): Regions depicting significant difference in the CT between CM and HC (A). Regions depicting significant difference between CM and HC in the correlation of average CT with HADS-A (B), HADS-D (C), and hours of sleep (D). A summary of the significant regions is reported in supplementary Table 2. All results are shown at *p* < 0.001 (uncorrected).

**Additional file 4 **: **Figure S3.** Episodic migraine patients (EM) and chronic migraine patients (CM): Regions depicting significant difference in the CT between EM and CM (A). Regions depicting significant difference between EM and CM in the regression of average CT with HADS-A (B), HADS-D (C), hours of sleep (D) and number of headache attacks per month (E). A summary of the significant regions is reported in Supplementary Table 3. All results are shown at *p* < 0.001 (uncorrected).

## Data Availability

The datasets generated and/or analyzed during the current study are not publicly available due to patient consent but could be available from the corresponding author on reasonable request and would be decided upon individual basis.

## Abbreviations

EM: Episodic Migraine
CM: Chronic Migraine
HC: Healthy Controls
SVR: Support Vector Regressions
CT: Cortical Thickness
fMRI: Functional magnetic resonance imaging
GMV: Grey matter Volume
DTI: Diffusion Tensor Imaging
MIDAS: Migraine Disability Assessment
HADS-A: Hamilton Anxiety Score
HADS-D: Hamilton Depression Score
MPRAGE: Magnetization Prepared Rapid Gradient Echo
NBS: Network Based Statistics
FOV: Field of View
DMN: Default Mode Network

## References

[1] Manzoni GC, Stovner LJ (2010). Epidemiology of headache. Handb Clin Neurol.

[2] Lipton RB, Bigal ME, Diamond M, Freitag F, Reed ML, Stewart WF (2007). Migraine prevalence, disease burden, and the need for preventive therapy. Neurology..

[3] Vos T, Flaxman AD, Naghavi M, Lozano R, Michaud C, Ezzati M (2012). Years lived with disability (YLDs) for 1160 sequelae of 289 diseases and injuries 1990-2010: a systematic analysis for the global burden of disease study 2010. Lancet..

[4] Headache Classification Committee of the International Headache Society (IHS) (2018). The International Classification of Headache Disorders, 3rd edition. Cephalalgia.

[5] Maleki N, Gollub RL (2016). What have we learned from brain functional connectivity studies in migraine headache?. Headache..

[6] Skorobogatykh K, van Hoogstraten WS, Degan D, Prischepa A, Savitskaya A, Ileen BM (2019). Functional connectivity studies in migraine: what have we learned?. J Headache Pain.

[7] Xu G, Cheng S, Qu Y, Cheng Y, Zhou J, Li Z (2020). The functional alterations in primary migraine: a systematic review and meta-analysis protocol. Medicine (Baltimore).

[8] Maleki N, Becerra L, Brawn J, Bigal M, Burstein R, Borsook D (2012). Concurrent functional and structural cortical alterations in migraine. Cephalalgia..

[9] Rocca MA, Ceccarelli A, Falini A, Colombo B, Tortorella P, Bernasconi L (2006). Brain gray matter changes in migraine patients with T2-visible lesions: a 3-T MRI study. Stroke.

[10] Liu J, Lan L, Li G, Yan X, Nan J, Xiong S (2013). Migraine-related gray matter and white matter changes at a 1-year follow-up evaluation. J Pain.

[11] Schmidt-Wilcke T, Ganssbauer S, Neuner T, Bogdahn U, May A (2008). Subtle grey matter changes between migraine patients and healthy controls. Cephalalgia..

[12] Lakhan SE, Avramut M, Tepper SJ (2013). Structural and functional neuroimaging in migraine: insights from 3 decades of research. Headache..

[13] Valfre W, Rainero I, Bergui M, Pinessi L (2008). Voxel-based morphometry reveals gray matter abnormalities in migraine. Headache..

[14] Jin C, Yuan K, Zhao L, Zhao L, Yu D, von Deneen KM (2013). Structural and functional abnormalities in migraine patients without aura. NMR Biomed.

[15] Mehnert J, May A. Functional and structural alterations in the migraine cerebellum. J Cereb Blood Flow Metab. 2017:271678X17722109 10.1177/0271678X17722109 PMC6446424 28737061

[16] Matharu MS, Good CD, May A, Bahra A, Goadsby PJ (2003). No change in the structure of the brain in migraine: a voxel-based morphometric study. Eur J Neurol.

[17] Mehnert J, Schulte L, May A (2020). No grey matter alterations in longitudinal data of migraine patients. Brain.

[18] Sheng L, Zhao P, Ma H, Yuan C, Zhong J, Dai Z (2020). A lack of consistent brain grey matter alterations in migraine. Brain.

[19] Kim J, Suh SI, Seol H, Oh K, Seo WK, Yu SW (2008). Regional grey matter changes in patients with migraine: a voxel-based morphometry study. Cephalalgia..

[20] Schmitz N, Admiraal-Behloul F, Arkink EB, Kruit MC, Schoonman GG, Ferrari MD (2008). Attack frequency and disease duration as indicators for brain damage in migraine. Headache..

[21] Schmitz N, Arkink EB, Mulder M, Rubia K, Admiraal-Behloul F, Schoonman GG (2008). Frontal lobe structure and executive function in migraine patients. Neurosci Lett.

[22] DaSilva AF, Granziera C, Snyder J, Hadjikhani N (2007). Thickening in the somatosensory cortex of patients with migraine. Neurology..

[23] Gaist D, Hougaard A, Garde E, Reislev NL, Wiwie R, Iversen P et al (2018) Migraine with visual aura associated with thicker visual cortex. Brain 10.1093/brain/awx382 29360944

[24] Granziera C, DaSilva AF, Snyder J, Tuch DS, Hadjikhani N (2006). Anatomical alterations of the visual motion processing network in migraine with and without aura. PLoS Med.

[25] Magon S, May A, Stankewitz A, Goadsby PJ, Schankin C, Ashina M (2019). Cortical abnormalities in episodic migraine: a multi-center 3T MRI study. Cephalalgia..

[26] Hougaard A, Amin FM, Hoffmann MB, Larsson HB, Magon S, Sprenger T (2015). Structural gray matter abnormalities in migraine relate to headache lateralization, but not aura. Cephalalgia..

[27] Schwedt TJ, Berisha V, Chong CD (2015). Temporal lobe cortical thickness correlations differentiate the migraine brain from the healthy brain. PLoS One.

[28] Sporns O (2013). Structure and function of complex brain networks. Dialogues Clin Neurosci.

[29] Koirala N, Fleischer V, Glaser M, Zeuner KE, Deuschl G, Volkmann J (2018). Frontal lobe connectivity and network community characteristics are associated with the outcome of subthalamic nucleus deep brain stimulation in patients with Parkinson’s disease. Brain Topogr.

[30] Bassett DS, Sporns O (2017). Network neuroscience. Nat Neurosci.

[31] Liu J, Zhao L, Li G, Xiong S, Nan J, Li J (2012). Hierarchical alteration of brain structural and functional networks in female migraine sufferers. PLoS One.

[32] Li K, Liu L, Yin Q, Dun W, Xu X, Liu J (2017). Abnormal rich club organization and impaired correlation between structural and functional connectivity in migraine sufferers. Brain Imaging Behav.

[33] Michels L, Villanueva J, O'Gorman RL, Muthuramam M, Koirala N, Buechler R (2019). Interictal hyperperfusion in the higher visual cortex in patients with episodic migraine. Headache.

[34] Hodkinson DJ, Veggeberg R, Wilcox SL, Scrivani S, Burstein R, Becerra L (2015). Primary somatosensory cortices contain altered patterns of regional cerebral blood flow in the Interictal phase of migraine. PLoS One.

[35] Headache Classification Committee of the International Headache S (2013). The international classification of headache disorders, 3rd edition (beta version). Cephalalgia..

[36] May A, Schulte LH (2016). Chronic migraine: risk factors, mechanisms and treatment. Nat Rev Neurol.

[37] Dodick DW, Turkel CC, DeGryse RE, Aurora SK, Silberstein SD, Lipton RB (2010). OnabotulinumtoxinA for treatment of chronic migraine: pooled results from the double-blind, randomized, placebo-controlled phases of the PREEMPT clinical program. Headache..

[38] Stewart WF, Lipton RB, Dowson AJ, Sawyer J (2001). Development and testing of the migraine disability assessment (MIDAS) questionnaire to assess headache-related disability. Neurology..

[39] Zigmond AS, Snaith RP (1983). The hospital anxiety and depression scale. Acta Psychiatr Scand.

[40] Dale AM, Fischl B, Sereno MI (1999). Cortical surface-based analysis. I. Segmentation and surface reconstruction. Neuroimage..

[41] Fischl B, Dale AM (2000). Measuring the thickness of the human cerebral cortex from magnetic resonance images. Proc Natl Acad Sci.

[42] Fischl B, Salat DH, Busa E, Albert M, Dieterich M, Haselgrove C (2002). Whole brain segmentation: automated labeling of neuroanatomical structures in the human brain. Neuron..

[43] Rosas HD, Liu AK, Hersch S, Glessner M, Ferrante RJ, Salat DH (2002). Regional and progressive thinning of the cortical ribbon in Huntington's disease. Neurology..

[44] Kuperberg GR, Broome MR, McGuire PK, David AS, Eddy M, Ozawa F (2003). Regionally localized thinning of the cerebral cortex in schizophrenia. Arch Gen Psychiatry.

[45] Reuter M, Schmansky NJ, Rosas HD, Fischl B (2012). Within-subject template estimation for unbiased longitudinal image analysis. Neuroimage..

[46] Rubinov M, Sporns O (2010). Complex network measures of brain connectivity: uses and interpretations. Neuroimage..

[47] Hosseini SM, Hoeft F, Kesler SR (2012). GAT: a graph-theoretical analysis toolbox for analyzing between-group differences in large-scale structural and functional brain networks. PLoS One.

[48] Fleischer V, Radetz A, Ciolac D, Muthuraman M, Gonzalez-Escamilla G, Zipp F et al (2019) Graph theoretical framework of brain networks in multiple sclerosis: a review of concepts. Neuroscience 403:35–53. 10.1016/j.neuroscience.2017.10.033 10.1016/j.neuroscience.2017.10.033 29101079

[49] Fleischer V, Koirala N, Droby A, Gracien R-M, Deichmann R, Ziemann U (2019). Longitudinal cortical network reorganization in early relapsing–remitting multiple sclerosis. Ther Adv Neurol Disord.

[50] Fleischer V, Groger A, Koirala N, Droby A, Muthuraman M, Kolber P (2017). Increased structural white and grey matter network connectivity compensates for functional decline in early multiple sclerosis. Mult Scler.

[51] Blondel VDG, Jean-Loup, Lambiotte R, Lefebvre E (2008). Fast unfolding of communities in large networks Journal of Statistical Mechanics. Theory Exp.

[52] Zalesky A, Fornito A, Bullmore ET (2010). Network-based statistic: identifying differences in brain networks. Neuroimage..

[53] Koirala N, Anwar AR, Ciolac D, Glaser M, Pintea B, Deuschl G (2019). Alterations in white matter network and microstructural integrity differentiate Parkinson's disease patients and healthy subjects. Front Aging Neurosci.

[54] Korgaonkar MS, Fornito A, Williams LM, Grieve SM (2014). Abnormal structural networks characterize major depressive disorder: a connectome analysis. Biol Psychiatry.

[55] Desikan RS, Ségonne F, Fischl B, Quinn BT, Dickerson BC, Blacker D (2006). An automated labeling system for subdividing the human cerebral cortex on MRI scans into gyral based regions of interest. Neuroimage..

[56] Bassett DS, Bullmore E, Verchinski BA, Mattay VS, Weinberger DR, Meyer-Lindenberg A (2008). Hierarchical organization of human cortical networks in health and schizophrenia. J Neurosci.

[57] He Y, Chen Z, Evans A (2008). Structural insights into aberrant topological patterns of large-scale cortical networks in Alzheimer's disease. J Neurosci.

[58] Achard S, Bullmore E (2007). Efficiency and cost of economical brain functional networks. PLoS Comput Biol.

[59] Bernhardt BC, Chen Z, He Y, Evans AC, Bernasconi N (2011). Graph-theoretical analysis reveals disrupted small-world organization of cortical thickness correlation networks in temporal lobe epilepsy. Cereb Cortex.

[60] Drucker H, Burges CJC, Kaufman L, Smola A, Vapnik V (1996). Support vector regression machines. Proceedings of the 9th International Conference on Neural Information Processing Systems.

[61] Riederer F, Schaer M, Gantenbein AR, Luechinger R, Michels L, Kaya M et al (2017) Cortical Alterations in Medication-Overuse Headache. Headache 57(2):255–265. 10.1111/head.12993. Epub 2016 Dec 2 10.1111/head.12993 28028803

[62] Messina R, Rocca MA, Colombo B, Valsasina P, Horsfield MA, Copetti M (2013). Cortical abnormalities in patients with migraine: a surface-based analysis. Radiology..

[63] Seifert CL, Magon S, Staehle K, Zimmer C, Foerschler A, Radue EW (2012). A case-control study on cortical thickness in episodic cluster headache. Headache..

[64] Vigano A, D'Elia TS, Sava SL, Auve M, De Pasqua V, Colosimo A (2013). Transcranial direct current stimulation (tDCS) of the visual cortex: a proof-of-concept study based on interictal electrophysiological abnormalities in migraine. J Headache Pain.

[65] Pohl H, Moisa M, Jung HH, Brenner K, Aschmann J, Riederer F et al (2020) Long-term effects of self-administered Transcranial direct current stimulation in episodic migraine prevention: results of a randomized controlled trial. Neuromodulation. 10.1111/ner.13292. Online ahead of print 10.1111/ner.13292 33078518

[66] Coppola G, Di Renzo A, Petolicchio B, Tinelli E, Di Lorenzo C, Serrao M et al (2020) Increased neural connectivity between the hypothalamus and cortical resting-state functional networks in chronic migraine. J Neurol 267(1):185–191. 10.1007/s00415-019-09571-y. Epub 2019 Oct 12 10.1007/s00415-019-09571-y 31606759

[67] Chen Z, Chen X, Liu M, Liu S, Shu S, Ma L (2016). Altered functional connectivity of the marginal division in migraine: a resting-state fMRI study. J Headache Pain.

[68] Liu J, Zhao L, Lei F, Zhang Y, Yuan K, Gong Q (2015). Disrupted resting-state functional connectivity and its changing trend in migraine suffers. Hum Brain Mapp.

[69] Hubbard CS, Khan SA, Keaser ML, Mathur VA, Goyal M, Seminowicz DA (2014). Altered Brain Structure and Function Correlate with Disease Severity and Pain Catastrophizing in Migraine Patients. eNeuro.

[70] Schwedt TJ, Schlaggar BL, Mar S, Nolan T, Coalson RS, Nardos B (2013). Atypical resting-state functional connectivity of affective pain regions in chronic migraine. Headache..

[71] Hadjikhani N, Ward N, Boshyan J, Napadow V, Maeda Y, Truini A (2013). The missing link: enhanced functional connectivity between amygdala and visceroceptive cortex in migraine. Cephalalgia..

[72] Michels L, Christidi F, Steiger VR, Sandor PS, Gantenbein AR, Landmann G et al (2017) Pain modulation is affected differently in medication-overuse headache and chronic myofascial pain - a multimodal MRI study. Cephalalgia 37(8):764–779. 10.1177/0333102416652625. Epub 2016 June 1 10.1177/0333102416652625 27250235

[73] Lee MJ, Park BY, Cho S, Kim ST, Park H, Chung CS (2019). Increased connectivity of pain matrix in chronic migraine: a resting-state functional MRI study. J Headache Pain.

[74] Riederer F, Marti M, Luechinger R, Lanzenberger R, von Meyenburg J, Gantenbein AR (2012). Grey matter changes associated with medication-overuse headache: correlations with disease related disability and anxiety. World J Biol Psychiatry.

[75] Neeb L, Bastian K, Villringer K, Israel H, Reuter U, Fiebach JB (2017). Structural Gray matter alterations in chronic migraine: implications for a progressive disease?. Headache..

