# Supplementary figures and images for: Structural brain network characteristics in patients with episodic and chronic migraine
Source: J Headache Pain. 2021 Mar 3;22(1):8. doi: 10.1186/s10194-021-01216-8 (PMC7927231; doi:10.1186/s10194-021-01216-8)

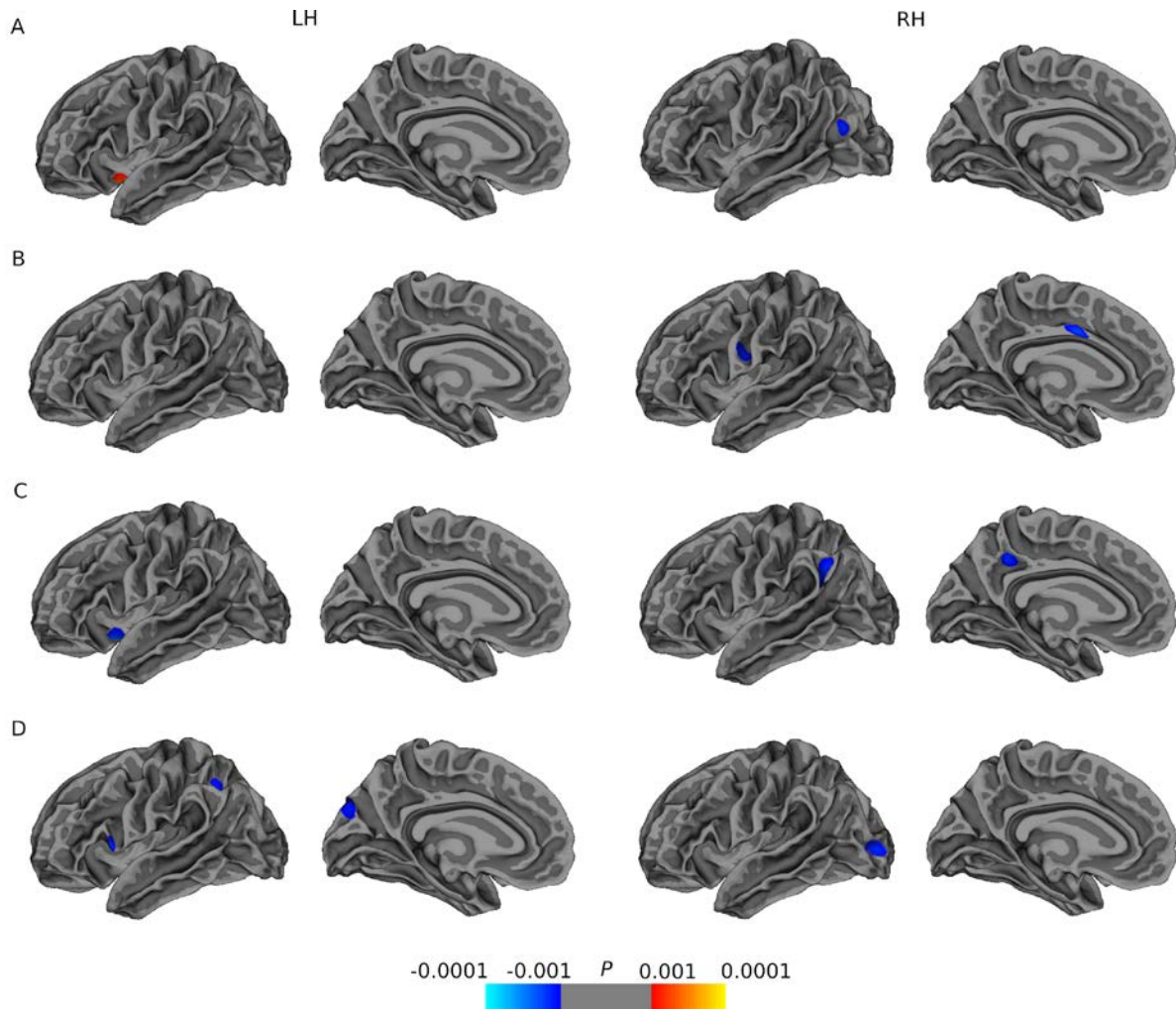

Supplement: Supplementary file 1 — Additional file 1 : Table S1. Summary of significant regions as shown in supplementary figure 1. Table S2. Summary of significant regions as shown in supplementary figure 2. Table S3. Summary of significant regions as shown in supplementary figure 3. [file 10194_2021_1216_MOESM3_ESM.pdf]

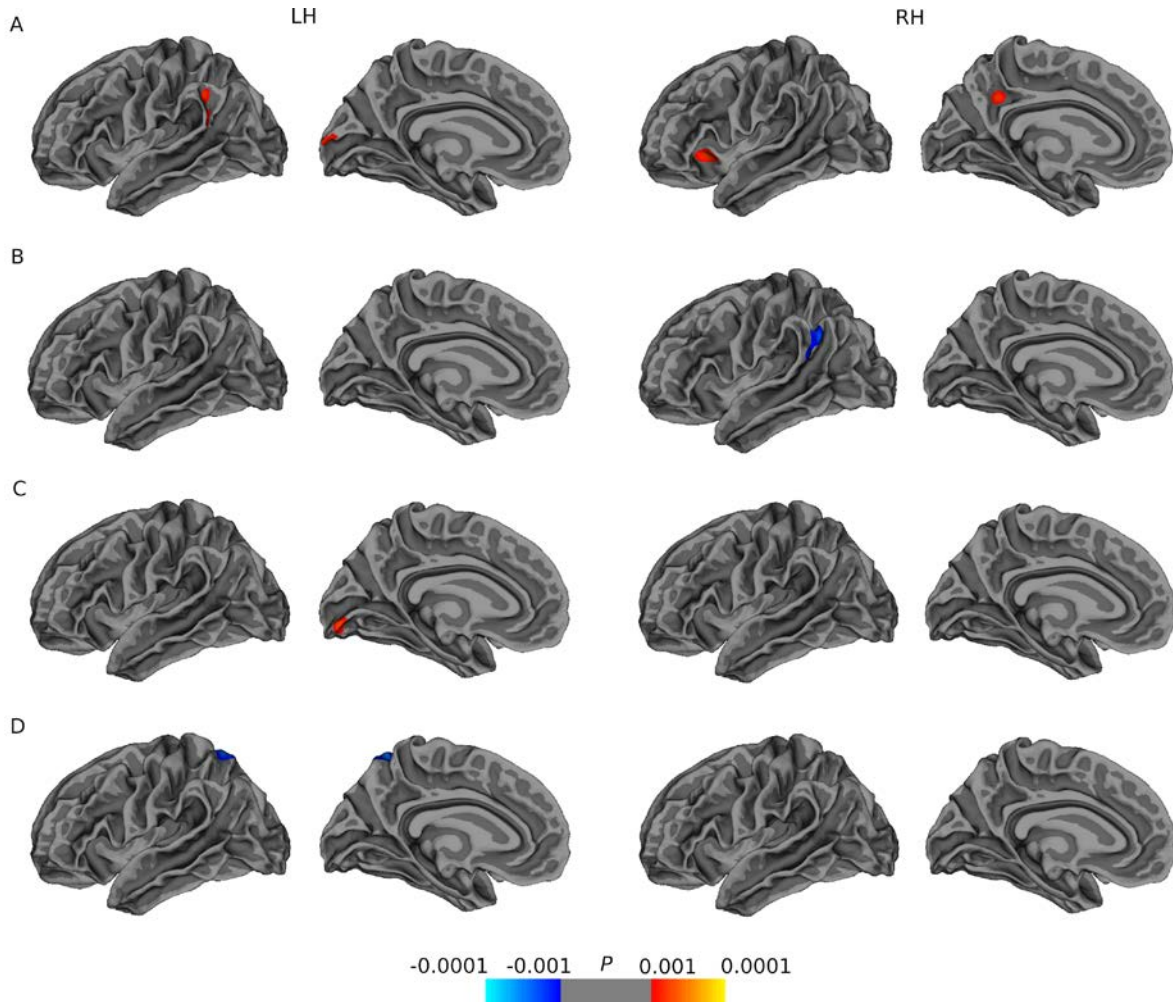

Supplement: Supplementary file 2 — Additional file 2 : Figure S1. Illustration of between-group differences for CT. (A) Regions depicting significant difference in the CT between EM and HC. Regions depicting significant difference between EM and HC in the correlation of average CT with HADS-A (B), HADS-D (C), and hours of sleep (D). A summary of the significant regions is reported in supplementary Table 1. All results are shown at p < 0.001 (uncorrected). [file 10194_2021_1216_MOESM2_ESM.pdf]

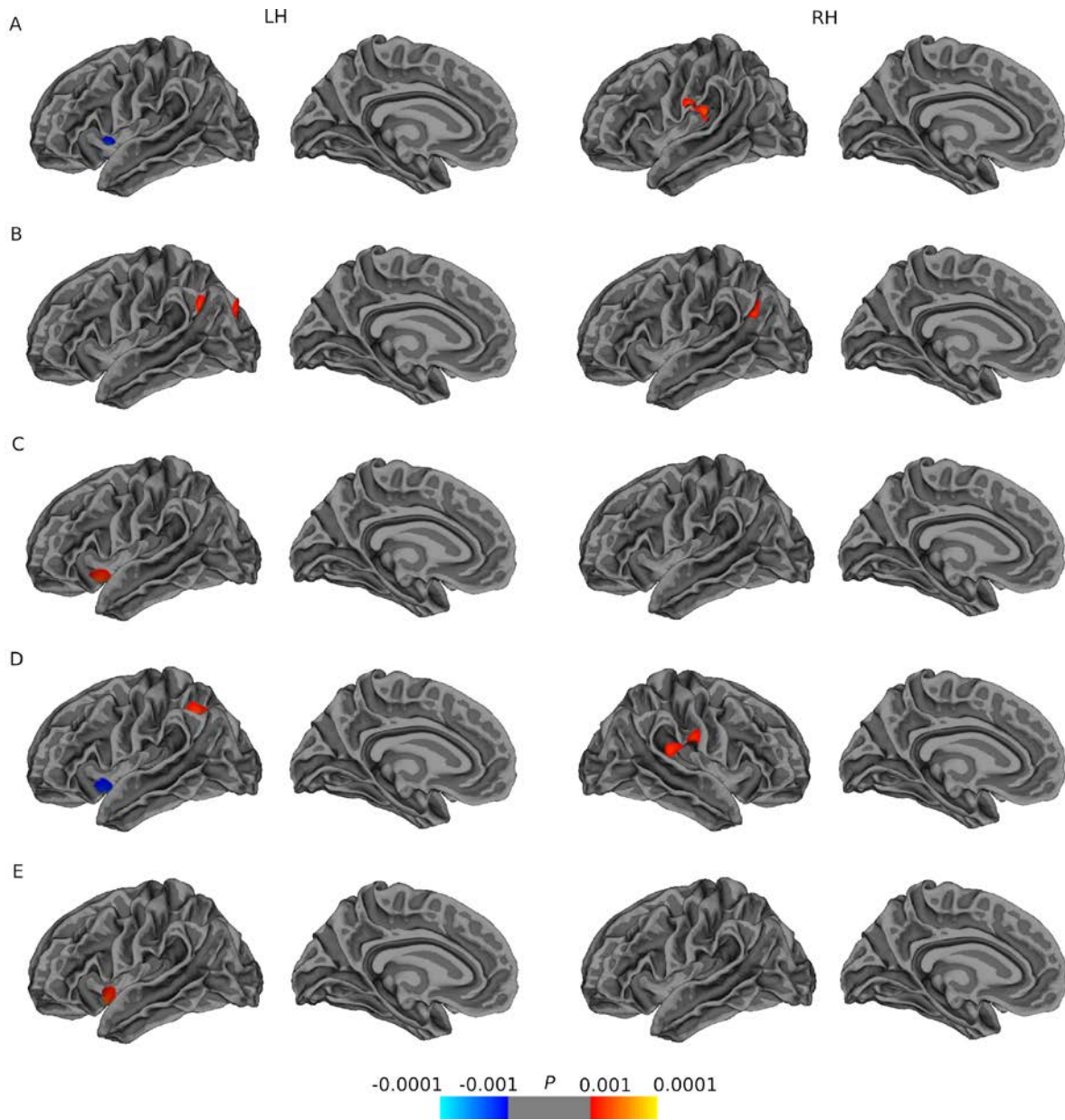

Supplement: Supplementary file 4 — Additional file 4 : Figure S3. Episodic migraine patients (EM) and chronic migraine patients (CM): Regions depicting significant difference in the CT between EM and CM (A). Regions depicting significant difference between EM and CM in the regression of average CT with HADS-A (B), HADS-D (C), hours of sleep (D) and number of headache attacks per month (E). A summary of the significant regions is reported in Supplementary Table 3. All results are shown at p < 0.001 (uncorrected). [file 10194_2021_1216_MOESM4_ESM.pdf]
