# Supplementary material for: Structural brain network characteristics in patients with episodic and chronic migraine
Source: J Headache Pain. 2021 Mar 3;22(1):8. doi: 10.1186/s10194-021-01216-8 (PMC7927231; doi:10.1186/s10194-021-01216-8)
Supplement: Supplementary file 3 — Additional file 3 : Figure S2. Chronic migraine patients (CM) and healthy controls (HC): Regions depicting significant difference in the CT between CM and HC (A). Regions depicting significant difference between CM and HC in the correlation of average CT with HADS-A (B), HADS-D (C), and hours of sleep (D). A summary of the significant regions is reported in supplementary Table 2. All results are shown at p < 0.001 (uncorrected). [file 10194_2021_1216_MOESM1_ESM.docx]

| Episodic Migraineurs - Healthy Controls | Region of Interest | -log_10_(p-value) | Size of cluster (mm^2^) |
| --- | --- | --- | --- |
| CT difference | Left lateral occipital | 4.0480 | 123.07 |
|  | Right Insula | 3.8627 | 39.57 |
|  | Right Lingual | 3.9254 | 20.55 |
|  | Right Precuneus | 3.2561 | 6.30 |
|  | Left Supramarginal | 3.3477 | 6.07 |
| Correlation with HADS-A | Right Precentral | -4.6023 | 37.81 |
| Correlation with HADS-D | Left Lingual | 3.4304 | 30.73 |
| Hours of Sleep | Left superior parietal | -5.0519 | 115.05 |

Supplementary Table 1: Summary of significant regions as shown in supplementary figure 1.

| Chronic Migraineurs - Healthy Controls | Region of Interest | -log_10_(p-value) | Size of cluster (mm^2^) |
| --- | --- | --- | --- |
| CT difference | Left inferior parietal | -3.6857 | 39.73 |
|  | Right lateral occipital | -3.2584 | 24.71 |
|  | Left Insula | 3.6973 | 6.96 |
|  | Left isthmus of cingulate gyrus | 3.1006 | 2.84 |
|  | Right inferior parietal | -3.0793 | 2.47 |
| Correlation with HADS-A | Right Caudal anterior cingulate | -3.6366 | 13.51 |
|  | Right Precentral | -3.2644 | 3.64 |
| Correlation with HADS-D | Left Insula | -4.2281 | 11.38 |
|  | Left posterior cingulate | -3.1789 | 6.51 |
|  | Right supramarginal | -3.0656 | 3.03 |
| Hours of Sleep | Left superior parietal | -3.8656 | 106.71 |
|  | Left pars opercularis | -3.9706 | 66.18 |
|  | Left insula | 3.2801 | 4.56 |
|  | Left isthmus cingulate | 3.1395 | 3.13 |

Supplementary Table 2: Summary of significant regions as shown in supplementary figure 2.

| Episodic - Chronic Migraineurs | Region of Interest | -log_10_(p-value) | Size of cluster (mm^2^) |
| --- | --- | --- | --- |
| CT difference | Right Supramarginal | 3.4560 | 19.34 |
|  | Right Insula | 3.5745 | 13.12 |
|  | Left inferior parietal | 3.4382 | 12.27 |
|  | Left Insula | -3.4388 | 6.09 |
|  | Right postcentral | 3.0888 | 2.51 |
| Correlation with HADS-A | Right inferior parietal | 3.2276 | 11.10 |
|  | Left superior parietal | 3.0724 | 4.28 |
| Correlation with HADS-D | Left superior parietal | 3.2836 | 11.15 |
|  | Left insula | 3.8540 | 11.07 |
| Hours of Sleep | Right supramarginal | 4.0789 | 57.40 |
|  | Right insula | 3.2023 | 6.74 |
|  | Right postcentral | 3.0683 | 2.25 |
|  | Left insula | -3.0616 | 2.0 |
| Number of attack (per month) | Left Insula | 3.8977 | 10.12 |

Supplementary Table 3: Summary of significant regions as shown in supplementary figure 3.
